# Community health volunteer support for regular blood pressure monitoring in Indonesia: spatial regression models

**DOI:** 10.1186/s41182-025-00765-x

**Published:** 2025-07-01

**Authors:** Mayumi Mizutani, Sofi Oktaviani, Harumi Bando, Heri Sugiarto, Ritsuko Nishide, Susumu Tanimura

**Affiliations:** 1 Department of Integrated Health Sciences, Nagoya University Graduate School of Medicine, Aichi, Japan; 2 Department of Public Health Nursing, Mie University Graduate School of Medicine, Mie, Japan; 3 Indramayu College of Health Science, Indramayu, West Java Indonesia; 4 Faculty of Nursing, School of Medicine, Nara Medical University, Nara, Japan; 5 Ngudi Waluyo University, Semarang, Central Java Indonesia; 6 Department of Nursing, Nagoya University of Arts and Sciences, Aichi, Japan

**Keywords:** Blood pressure monitoring, Hypertension, Community health workers, Volunteers, Spatial regression, Indonesia, Resource-limited settings

## Abstract

**Background:**

Community-based blood pressure monitoring is essential for effective hypertension management, especially in low- and middle-income countries like Indonesia, where community health volunteers (CHVs) are vital. However, there is a lack of strong evidence regarding which aspects of CHV support are most effective at encouraging regular blood pressure monitoring. This study examined the spatially adjusted relationship between the prevalence of regular blood pressure monitoring and the specific traits of Indonesian CHV support.

**Methods:**

The researchers conducted an ecological study utilizing sub-district level data from the fifth wave of the Indonesian Family Life Survey. The analysis included data on 25,829 individuals across 1774 sub-districts, with a focus on 612 CHVs in 259 sub-districts. To explore the spatially adjusted relationships between regular blood pressure monitoring and 16 CHV traits, ordinary regression and spatial regression models were employed. Additionally, geographically weighted regression (GWR) was implemented to examine geographical variations in the strength of these associations.

**Results:**

The overall prevalence of regular blood pressure monitoring across the 1774 sub-districts was 17.5%. Regression models revealed positive associations between this prevalence and CHVs being described as “considerate and kind” (B = [3.85, 4.24], p = 0.038–0.048) and “helpful” (B = [4.60, 4.82], p = 0.038–0.041). The GWR analysis showed notable variations in regression coefficients, with “considerate and kind” yielding B = [3.79, 4.07] and “helpful” yielding B = [4.42, 4.79], both demonstrating stronger associations in the western and northern region of Sumatra Island. Meanwhile, “values artistic/aesthetic experiences” showed a negative association, significant only in spatial regression models (B = [− 2.47, − 2.44], p = 0.046–0.048).

**Conclusions:**

This study emphasized the crucial role that CHVs play in promoting regular blood pressure monitoring in Indonesia. Compassion, kindness, and helpfulness were especially vital for improving community-based blood pressure monitoring, which leads to the better management of hypertension.

## Background

High blood pressure, or hypertension, has become a major global health issue, especially in low- and middle-income countries (LMICs). In the past 30 years, the number of people aged 30–79 who are affected by high blood pressure has doubled from 648 million to 1.28 billion, with most cases occurring in LMICs [1, 2]. In Indonesia, the prevalence of hypertension among individuals aged 18 and above has surged from 25.8% in 2013 to 30.8% in 2023 [3, 4]. Effective strategies for preventing and controlling hypertension are urgently required, especially in resource-limited settings. 

Community-based blood pressure monitoring is essential for preventing and managing hypertension in resource-limited settings, where community health volunteers (CHVs) play a crucial role. Numerous systematic reviews have emphasized the importance of home and primary health care initiatives in enhancing blood pressure management [5–9]. Despite this, many individuals with elevated blood pressure in LMICs remain undiagnosed; only 73.6% of individuals report having ever had their blood pressure measured [10]. The consequences of undiagnosed and uncontrolled hypertension can be severe, leading to increased mortality and morbidity from conditions such as kidney disease, heart disease, and stroke [11, 12]. Additionally, these health issues contribute to rising healthcare costs [13] and lost productivity [14].

In Indonesia, community health posts known as *posyandu lansia* or *posbindu* play a vital role in blood pressure monitoring and health education. These posts, which are managed by healthcare professionals like nurses and CHVs, locally called *kader kesehatan*, provide monthly opportunities for individuals to have their blood pressure checked and receive essential health information [15, 16]. There is global evidence that CHVs are instrumental in promoting health in communities, including blood pressure management behaviors [17–21]. Nevertheless, there is also a significant lack of research on which specific traits of CHVs are most effective in promoting regular blood pressure monitoring among community members.

Spatial analysis can improve our understanding of true associations based on spatial dependence. Tobler’s first law of geography asserts that “everything is related to everything else, but near things are more related than distant things” [22]. For example, a sub-district that shows a strong correlation between regular blood pressure monitoring and CHVs’ support will likely be situated near other sub-districts with similar correlations. To accurately evaluate these relationships, spatial regression models can be utilized to account for spatial effects [23], as traditional regression models may yield invalid results by overlooking these dependencies. Moreover, spatial analysis can illuminate regional variations in the strength of associations. While the traditional ordinary least squares (OLS) model assumes that coefficients remain constant across a studied area, geographically weighted regression (GWR) accounts for possible spatial variation in these relationships [24]. For instance, some sub-districts will exhibit stronger links between regular blood pressure monitoring and support from CHVs, while others will show weaker connections. GWR is especially effective for calculating locally specific coefficients that capture these variations.

This study aimed to: (1) map the geographic distribution of regular blood pressure monitoring prevalence across sub-districts in Indonesia, (2) identify the traits of CHVs associated with this prevalence, and (3) clarify the regional variations in the strength of these associations. The findings highlight the most effective CHV support methods that enhance regular blood pressure monitoring at the community level, providing valuable insights for community-based intervention strategies for preventing and controlling hypertension.

## Methods

### Data source and study population

This ecological study utilized data from the fifth wave of the Indonesian Family Life Survey (IFLS-5), which was conducted in 2014–2015. This data was downloaded on December 25, 2021. The IFLS is an ongoing longitudinal survey initiated in 1993–1994, with subsequent waves conducted in 1997–1998, 2000, 2007–2008, and 2014–2015 by RAND Corporation and Survey Meter. This survey offers comprehensive information on Indonesian demographics, life, and health indicators at the individual level and is representative of approximately 83% of the Indonesian population in 13 out of 27 provinces [25]. This study focused on individuals aged 15 and above. The researchers collected individual-level data, which were linked and aggregated at the sub-district level for analysis, using the sub-district as the unit of analysis. Additionally, geospatial vector data was obtained on May 5, 2022 [26].

### Variables and data process

The objective variable measured was the percentage of respondents in each sub-district who reported having their blood pressure checked regularly. This was assessed with the question, “How regularly have you had your blood pressure checked?” Respondents could answer with either “regularly” or “irregularly.” The percentage of individuals who answered “regularly” was then calculated for each sub-district.

The explanatory variables comprised 16 CHV traits. In this study, CHVs were defined as individuals who answered “Yes” to the question, “During the last 12 months, did you participate in or use *posyandu lansia* (community health posts)?” and selected “labor/time” from the available options (“labor/time,” “money,” “goods”). The mean values of the 16 traits were calculated for each sub-district. Each trait was indicated by the respondents’ level of agreement, measured on Likert scales from 1 (“strongly disagree”) to 5 (“strongly agree”) for items 1–15, and from 1 (“strongly agree”) to 4 (“strongly disagree”) for item 16. To ensure consistency, the scale was reversed for item 16, which aligned it with items 1–15. The 16 traits and their related items were as follows:

1. Talkative: “I see myself as someone who is talkative;”
2. Thorough: “I see myself as someone who does a thorough job;”
3. Original and innovative: “I see myself as someone who is original and comes up with new ideas;”
4. Reserved: “I see myself as someone who is reserved;”
5. Relaxed: “I see myself as someone who is relaxed and handles stress well;”
6. Forgiving: “I see myself as someone who has a forgiving nature;”
7. Anxious: “I see myself as someone who worries a lot;”
8. Imaginative: “I see myself as someone who has an active imagination;”
9. Lazy: “I see myself as someone who tends to be lazy;”
10. Aesthetic: “I see myself as someone who values artistic and aesthetic experiences;”
11. Considerate and kind: “I see myself as someone who is considerate and kind to almost everyone;”
12. Efficient: “I see myself as someone who does things efficiently;”
13. Outgoing and sociable: “I see myself as someone who is outgoing and sociable;”
14. Rude: “I see myself as someone who is sometimes rude to others;”
15. Nervous: “I see myself as someone who gets nervous easily;”
16. Helpful: “I see myself as someone who is willing to help people in this village if they need it.”

Adjustment variables included gender, age, education, marital status, economic status, hypertension diagnosis, and elevated blood pressure. Previous studies have indicated that individuals who are older, live in urban areas, are more educated, and have received a hypertension diagnosis tend to monitor their blood pressure more frequently [27–30]. To assess these variables, the percentage of women and the mean age in each sub-district was calculated. Education was determined by asking individuals about the highest level of education they had completed, and the percentage of individuals who attended junior high school or above was calculated for each sub-district. Marital status was assessed by dividing the number of married respondents by the total number of respondents and multiplying by 100 to obtain a percentage for each sub-district. Economic status was evaluated by asking individuals to envision a six-step ladder, with one representing the poorest and six the richest, and to indicate which step they belonged to. The responses, coded from one to six, were then used to calculate the mean economic status for each sub-district.

Hypertension diagnosis was determined based on whether individuals answered “Yes” to the question, “Has a doctor, paramedic, nurse, or midwife ever told you that you had hypertension?” and indicated “doctor” when asked, “Who diagnosed the hypertension condition?” The percentage of individuals diagnosed with hypertension was calculated for each sub-district. Raised blood pressure was defined using respondents’ three blood pressure measurements. The mean systolic and diastolic blood pressure was calculated from these three measurements. An Omron HEM-7203 device was used for blood pressure measurement; typically, a standard-sized cuff was employed, with larger cuffs available if needed [25]. Individuals were classified as having raised blood pressure if their mean systolic blood pressure was 140 mm of mercury (mmHg) or higher and/or their mean diastolic blood pressure was 90 mmHg or higher. Finally, the percentage of individuals with raised blood pressure was calculated for each sub-district.

### Statistical analyses and visualization

From the dataset of individuals (n = 51,731), those with missing data on regular blood pressure monitoring (n = 25,902) were excluded. The final analysis included data from 25,829 individuals aged 15 and above across 1774 sub-districts in 24 provinces, including 612 CHVs in 259 of those sub-districts in 18 provinces.

First, the descriptive statistics for all variables were calculated at the sub-district level. Additionally, choropleth maps were created to depict the geographic distribution of regular blood pressure monitoring across the 1774 sub-districts. These maps were crucial for fully understanding the regional variations in the availability of regular blood pressure monitoring.

Next, an OLS model was applied to assess the relationship between the prevalence of regular blood pressure monitoring and CHV traits across 259 sub-districts while controlling for relevant adjustment variables. The variance inflation factor (VIF) was calculated to identify any potential multicollinearity issues among the explanatory variables. Additionally, the adequacy of the sample size was evaluated for addressing the research question by calculating the statistical power of the models [31] using the effect size f^2^ [32].

Spatial regression models were employed to identify spatially adjusted factors using a k-nearest neighbor approach. Based on Tobler’s first law of geography [22], it was suspected that spatial autocorrelation was present, meaning that nearby sub-districts could influence both objective and explanatory variables. The five geographically closest sub-districts to each sub-district were defined as that sub-district’s neighbors. Because the extent of spatial dependency was unknown prior to the analysis, various spatial regression models were fitted to the data, including the spatial autoregressive model (SAR), spatial error model (SEM), and spatial lag model (SLM) [33]. The models were assessed using Akaike’s information criterion (AIC), with a lower value indicating a better fit.

To evaluate the geographical variation in the regression coefficients between the prevalence of regular blood pressure monitoring and CHV traits, a GWR was conducted. Statistical significance was determined by a *p*-value of < 0.05. All the data processing and statistical analyses were performed using R version 4.4.1 [34]. Spatial analysis was carried out with the following packages: spatialreg version 1.3–6 [35], and spgwr version 0.6–37 [36].

## Results

### Sub-district characteristics

**Fig. 1:**
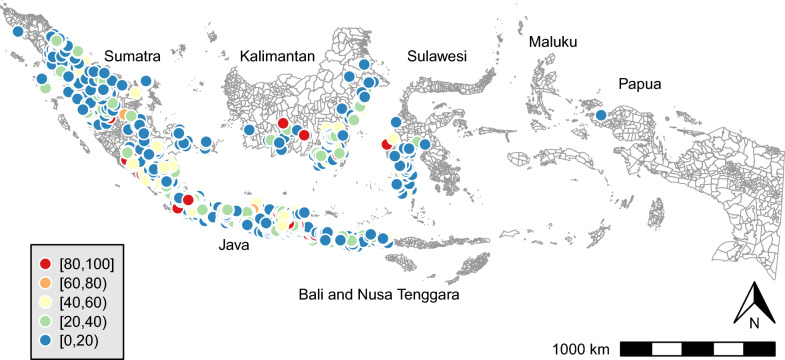
Prevalence of regular blood pressure monitoring in sub-districts (%, n = 1774)

Table 1 presents the descriptive characteristics of the sub-district population. The mean age was 35.03 years (standard deviation [SD]: 8.07), with the majority being female (58.41%). On average, 78.86% had attended junior high school. The mean prevalence of diagnosed hypertension was 6.81%, while the mean prevalence of raised blood pressure measured during assessments was 19.10%. The mean prevalence of regular blood pressure monitoring was 17.51%. Figure 1 illustrates the spatial variation of this prevalence across the 1774 sub-districts. Notably, 4.0% of these sub-districts (71 out of 1774) reported a 100% prevalence of regular blood pressure monitoring.

**Table 1:** Sub-district demographic characteristics

	n	Mean	SD	Min	Max
Women (%)	1774	58.41	24.23	0	100
Mean age (year)	1774	35.03	8.07	15	84
Attended junior high school (%)	1774	78.86	28.31	0	100
Married (%)	1774	76.94	29.32	0	100
Economic status	1774	3.09	0.65	1	6
Hypertension diagnosis (%)	1774	6.81	14.51	0	100
Raised blood pressure (%)	1774	19.10	24.19	0	100
Regular blood pressure monitoring (%)	1774	18.16	24.43	0	100
*CHVs’ traits*					
1. Talkative	259	3.26	0.97	1	5
2. Thorough	259	4.19	0.50	2	5
3. Original and innovative	259	3.64	0.83	1	5
4. Reserved	259	2.88	0.97	1	5
5. Relaxed	259	3.98	0.58	2	5
6. Forgiving	259	4.18	0.58	1	5
7. Anxious	259	3.21	0.98	1	5
8. Imaginative	259	3.56	0.85	1	5
9. Lazy	259	2.40	0.79	1	5
10. Aesthetic	259	4.00	0.69	1	5
11. Considerate and kind	259	4.17	0.48	2	5
12. Efficient	259	3.84	0.67	1.3	5
13. Outgoing and sociable	259	4.20	0.57	2	5
14. Rude	259	2.55	0.84	1	5
15. Nervous	259	2.71	0.90	1	5
16. Helpful	259	3.21	0.36	2	4

The means represent percentages for categorical variables such as women, attended junior high school, married, hypertension diagnosis, raised blood pressure, and regular blood pressure monitoring. For continuous variables such as age, economic status, and CHV’s traits, the means reflect actual average values

Economic status: mean values for each sub-district are scaled from 1 (poorest) to 6 (richest); hypertension diagnosis: the percentage of respondents who reported being diagnosed with hypertension by a doctor; raised blood pressure: the percentage of respondents with elevated blood pressure levels, defined as having a mean systolic blood pressure of ≥ 140 mmHg and/or a mean diastolic blood pressure of ≥ 90 mmHg, based on the average of three measurements; regular blood pressure monitoring: the percentage of respondents who answered “regularly” to the question “How regularly have you had your blood pressure checked?” for each sub-district; CHVs: community health volunteers; CHV traits: mean values of 16 traits assessed for CHVs in each sub-district, measured using a scale of 1 (strongly disagree) to 5 (strongly agree) for items 1–15, and 1 (strongly disagree) to 4 (strongly agree) for item 16

On average, there were 2.36 CHVs per sub-district, with a range of 1–14 volunteers. Of the CHVs, 73.37% (449 of 612) were female, with a mean age of 53.10 years (SD: 14.47). Of the 16 traits evaluated for CHVs, the highest average score using the five-point Likert scale was given to “outgoing and sociable” (4.20), closely followed by “thorough” (4.19), “forgiving” (4.18), “considerate and kind” (4.17), and “aesthetic” (4.00). The “helpful” trait received a mean score of 3.21 on the four-point Likert scale, which is approximately equivalent to a mean score of 4.01 on a five-point Likert scale.

### Association between regular blood pressure monitoring and CHVs’ traits

Table 2 presents the OLS results and spatial regression models that illustrate the relationships between the prevalence of regular blood pressure monitoring at the sub-district level and CHVs’ traits. The study identified a statistically significant positive association between blood pressure monitoring prevalence and the traits “considerate and kind” (B = [3.85, 4.24], *p* = 0.038–0.048) and “helpful” (B = [4.60, 4.82], *p* = 0.038–0.041). The strength of association for these two traits was similar, with “considerate and kind” (β = 0.15) and “helpful” (β = 0.13). VIF values ranged from 1.11 to 1.52, indicating no significant multicollinearity issues among the explanatory variables. The model’s statistical power (1−β) was 0.85, suggesting that the sample size was sufficient for addressing the research question. Conversely, the “Aesthetic” trait exhibited a negative association with the prevalence of regular blood pressure monitoring, which was only evident in the spatial regression models (B = [− 2.47, − 2.44], *p* = 0.046–0.048). The best-fit model was the OLS, which had the lowest AIC value of 2056.8.

**Table 2:** Association between regular blood pressure monitoring and CHVs’ traits

	OLS			SAR		SEM		SLM	
	B	β	*p*	B	*p*	B	*p*	B	*p*
CHVs’ traits									
1. Talkative	0.35	0.03	0.720	0.50	0.596	0.50	0.596	0.45	0.635
2. Thorough	− 1.69	− 0.06	0.383	− 1.28	0.486	− 1.28	0.486	− 1.45	0.431
3. Original and innovative	0.59	0.04	0.599	0.54	0.619	0.54	0.619	0.54	0.615
4. Reserved	− 0.43	− 0.03	0.653	− 0.16	0.862	− 0.16	0.862	− 0.20	0.830
5. Relaxed	1.24	0.05	0.441	1.14	0.458	1.14	0.458	1.17	0.443
6. Forgiving	− 0.88	− 0.04	0.604	− 0.66	0.681	− 0.66	0.681	− 0.66	0.683
7. Anxious	− 0.95	− 0.07	0.296	− 0.84	0.332	− 0.84	0.332	− 0.86	0.322
8. Imaginative	− 0.27	− 0.02	0.797	− 0.23	0.821	− 0.23	0.821	− 0.23	0.819
9. Lazy	− 1.32	− 0.08	0.257	− 1.73	0.136	− 1.73	0.136	− 1.68	0.145
10. Aesthetic	− 2.51	− 0.13	0.054	− **2.47**	**0.046**	− **2.47**	**0.046**	− **2.44**	**0.048**
11. Considerate and kind	**4.24**	**0.15**	**0.038**	**3.85**	**0.048**	**3.85**	**0.048**	**3.95**	**0.043**
12. Efficient	− 2.02	− 0.10	0.131	− 2.10	0.102	− 2.10	0.102	− 2.06	0.107
13. Outgoing and sociable	− 2.10	− 0.09	0.231	− 1.91	0.257	− 1.91	0.257	− 1.91	0.255
14. Rude	1.01	0.06	0.345	1.11	0.287	1.11	0.287	1.12	0.284
15. Nervous	− 0.25	− 0.02	0.810	− 0.02	0.983	− 0.02	0.983	− 0.05	0.960
16. Helpful	**4.82**	**0.13**	**0.041**	**4.60**	**0.039**	**4.60**	**0.039**	**4.63**	**0.038**
Adjustment variables									
Women (%)	0.07	0.06	0.330	0.05	0.452	0.05	0.452	0.05	0.482
Mean age (year)	− 0.06	− 0.02	0.782	− 0.08	0.681	− 0.08	0.681	− 0.09	0.667
Attended junior high (%)	0.05	0.06	0.360	0.05	0.258	0.05	0.258	0.05	0.274
Married (%)	**0.15**	**0.17**	**0.010**	**0.15**	**0.012**	**0.15**	**0.012**	**0.14**	**0.013**
Economic status	**6.17**	**0.17**	**0.020**	**5.98**	**0.018**	**5.98**	**0.018**	**5.86**	**0.020**
Hypertension diagnosis (%)	0.16	0.10	0.148	0.16	0.149	0.16	0.149	0.16	0.133
Raised blood pressure (%)	0.06	0.07	0.365	0.05	0.441	0.05	0.440	0.05	0.437
λ				0.08	0.485	0.08	0.485		
ρ								0.10	0.305
Adjusted R^2^	0.08								
F statistic (df = 23; 233)	1.95		**0.007**						
AIC	2056.8			2058.4		2058.4		2057.8	
f^2^	0.09								
1 − β	0.85								

OLS: ordinary least squares model; SAR: spatial autoregressive model; SEM: spatial error model; SLM: spatial lag model; AIC: Akaike’s information criterion. Boldface indicates statistical significance

Objective variable: Regular blood pressure monitoring, defined as the percentage of individuals who answered “Regularly” to the question, “How regularly have you had your blood pressure checked?” for each sub-district

Explanatory variables: The mean values of 16 community health volunteer (CHV) traits in each sub-district, gauged on a scale of 1 (strongly disagree) to 5 (strongly agree) for items 1–15, and 1 (strongly disagree) to 4 (strongly agree) for item 16

Adjustment variables: Mean age, percentage of women, percentage of individuals who attended junior high or above, percentage of married individuals, mean economic status scaled from 1 (poorest) to 6 (richest), percentage of individuals diagnosed with hypertension by a doctor, and percentage of respondents with raised blood pressure levels, defined as systolic blood pressure ≥ 140 mmHg and/or diastolic blood pressure ≥ 90 mmHg

### Regional variations

**Fig. 2:**
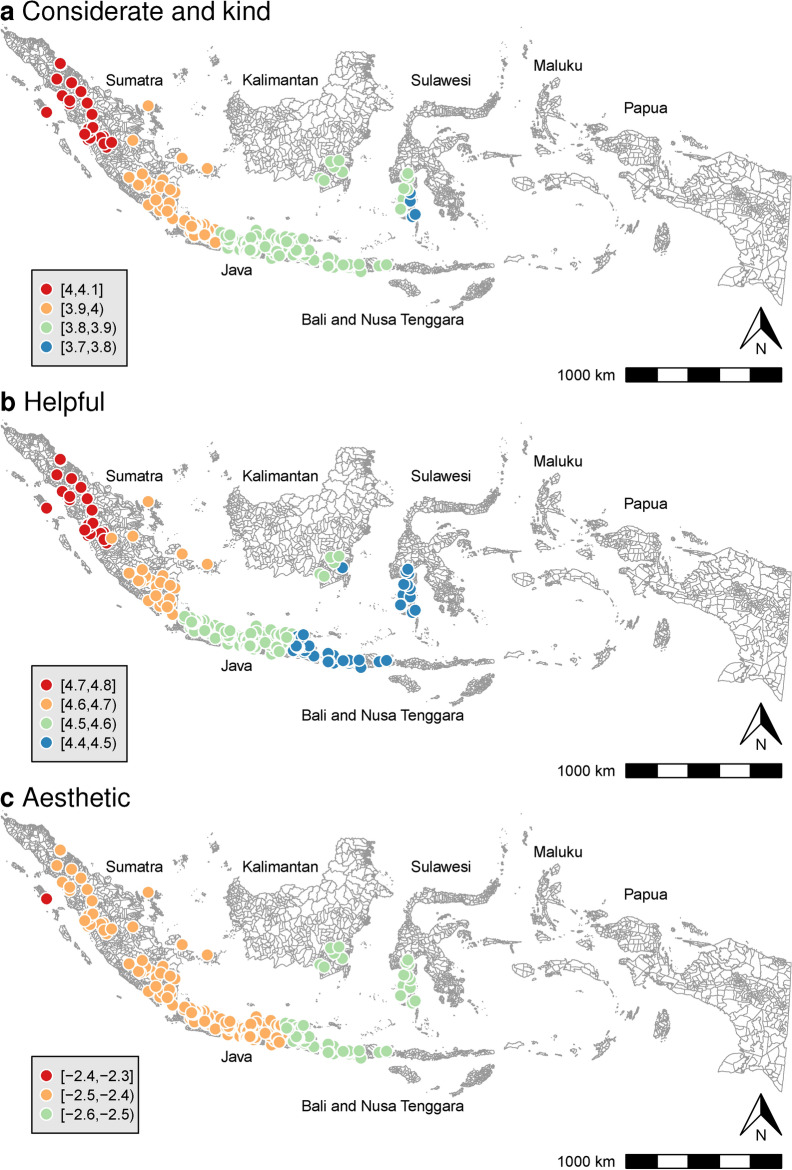
Regression coefficients of CHV traits against regular blood pressure monitoring. Note: CHV = community health volunteers. This figure illustrates the local regression coefficients of CHV traits in relation to the prevalence of regular blood pressure monitoring, using geographically weighted regression (GWR) after adjusting for covariates. Considerate and kind: Mean response of CHVs to the statement, “I see myself as someone who is considerate and kind to almost everyone;” Helpful: Mean response of CHVs to the statement, “I see myself as someone who is willing to help people in this village if they need it;” Aesthetic: Mean response of CHVs to the statement, “I see myself as someone who values artistic/aesthetic experiences”

Figure 2 uses GWR to illustrate the local regression coefficients for each CHV trait in relation to the prevalence of regular blood pressure monitoring, after accounting for adjustment variables and other CHV traits. The association strength for “considerate and kind” showed geographical variation (B = [3.79, 4.07]), with stronger correlations found in the western and northern regions of Sumatra Island (Fig. 2a). “Helpful” also exhibited geographical variation (B = [4.42, 4.79]), with stronger associations in the western and northern region of Sumatra Island (Fig. 2b). In contrast, the “aesthetic” trait (Fig. 2c) showed minimal geographical variation (B = [− 2.55, − 2.40]).

## Discussion

This study investigated spatial variations in the prevalence of regular blood pressure monitoring and its association with CHV support at the sub-district level in Indonesia. Notably, a significant discrepancy was found between the prevalence of diagnosed hypertension (6.81%) and the prevalence of elevated blood pressure (19.10%). This indicates that a substantial number of individuals may have undiagnosed hypertension. This is consistent with 2023 data from the Indonesian Ministry of Health, which reported a similar trend in which the percentage of diagnosed hypertension was lower than the prevalence of elevated blood pressure measured (5.9% vs. 26.0%) among individuals aged 18–59 [4]. This gap can be attributed to several factors, including limited access to blood pressure monitoring device [37], a lack of awareness among the population about the risks of hypertension and the importance of regular monitoring [38], and affordability and accessibility barriers within the healthcare system [39]. The analysis showed that the mean prevalence of regular blood pressure monitoring across the 1774 sub-districts was only 17.51%. The Indonesian Ministry of Health recommends that individuals without the risk of noncommunicable diseases have their blood pressure checked at least once every 3 months [40]. There is therefore an urgent need to promote regular blood pressure monitoring within the population. This study’s findings also revealed that only 4% of the sub-districts exhibited a higher prevalence of regular blood pressure monitoring. Further investigation into these sub-districts could yield valuable insights, as healthcare practitioners may have adopted effective strategies to encourage regular blood pressure monitoring.

To promote regular blood pressure monitoring in a community, this study identified three key CHV traits that influence this behavior in patients: being considerate and kind, being helpful, and not focusing on aesthetics. There was a positive correlation between being considerate, kind, and helpful and the frequency of blood pressure monitoring. In sub-districts in which CHVs demonstrated high levels of these traits, there is a significant increase in the number of individuals who regularly check their blood pressure. This association was particularly notable in the western and northern regions of Sumatra Island. In the western region, the Minangkabau people—the largest matrilineal society in the world—have a unique cultural structure that values women’s opinions and emphasizes their essential contributions to decision-making processes [41–43]. Since the majority of CHVs in this study were women (73.37%), those in western Sumatra were likely to be respected and influential within their communities, benefiting from the esteemed role of women in Minangkabau culture. This cultural respect likely contributed significantly to the observed increase in regular blood pressure monitoring among community members. Additionally, Minangkabau women are often characterized as compassionate [41]. In northern Sumatra, where the Batak ethnicity predominates, a cultural belief emphasizes the importance of kindness. The Batak people tend to strongly believe that good deeds attract blessings from God [44]. These cultural traits likely led to the result of CHVs in those areas being more likely to exhibit kindness and helpfulness.

Conversely, a negative association was found between the “aesthetic” trait and the frequency of blood pressure monitoring. In sub-districts where CHVs exhibited lower levels of this trait, there was a significant increase in the prevalence of blood pressure monitoring. CHVs are crucial for the prevention and management of hypertension, especially in resource-limited settings, as they provide lifestyle counseling and help facilitate access to healthcare facilities [18]. Rather than focusing exclusively on artistic and aesthetic experiences, it is important to emphasize the supportive care provided by CHVs that encourages individuals to monitor their blood pressure regularly. Additionally, this association was identified solely through spatial regression models, indicating that traditional OLS analysis may underestimate its impact. It is therefore vital to utilize both OLS and spatial regression models to fully understand the associations.

Research has demonstrated that patient-centered communication can greatly improve treatment adherence in hypertensive patients [45]. In Indonesia’s primary healthcare system, CHVs and nurses play a crucial role in providing community-based blood pressure monitoring services. This is especially important in rural areas like western Sumatra, where population density is low and the health workforce is more limited compared to the national targets [46–48]. In such contexts, CHVs can have a significant impact because they actively engage with community members and invite them to utilize health services. Empowering CHVs to be not only considerate and helpful but also focused on promoting health would encourage community members to adopt health-promoting behaviors like regular blood pressure checks. Currently, training programs primarily emphasize enhancing CHVs’ knowledge [49, 50]. CHV training program in Riau, which is located on Sumatra Island, includes effective communication skills [50]. To enhance the frequency of blood pressure monitoring, it is crucial to incorporate the three key traits into CHV training curricula across Indonesia.

This study’s strength was in its detailed examination of the traits of CHVs who provide effective support for regular blood pressure monitoring. These findings offer valuable insights for developing further intervention strategies that would empower CHVs to improve community-based blood pressure monitoring. However, the study also had several limitations. As an ecological study, it may be vulnerable to ecological fallacies [51]. Future research that incorporates individual-level data is essential for better determining whether the associations observed at the sub-district level are applicable to individuals. Such studies would support the development of evidence-based interventions aimed at improving local blood pressure monitoring strategies. Additionally, the study’s definition of “regular blood pressure monitoring” relied solely on participants’ perceptions. Future studies should gather individual data on the actual frequency of blood pressure monitoring to achieve a more accurate assessment. The responses regarding CHV traits were also based on participants’ perceptions, which could introduce social desirability bias into the findings. Finally, the analysis was based on data from 1774 sub-districts concerning blood pressure monitoring and 259 sub-districts regarding CHV information, which may limit the generalizability of these findings to other communities. Therefore, future research should explore a broader range of contexts to better understand variations in both practices and outcomes.

## Conclusions

This ecological study investigated the relationship between CHV traits and the regularity of blood pressure monitoring in Indonesia while taking spatial adjustments into account. The three key traits identified as having the most important impact on encouraging blood pressure monitoring were being “considerate and kind,” “helpful,” and not prioritizing “aesthetic experiences.” Notably, these associations were strongest in the western and northern region of Sumatra Island. To leverage these insights, policy efforts should focus on training CHVs to develop and strengthen these characteristics. By fostering these traits through targeted training and establishing supportive collaborations with public health nurses, Indonesia can enhance community-based hypertension prevention efforts. These findings illustrated the significance of specific CHV traits for promoting effective community-based blood pressure monitoring initiatives. By harnessing, encouraging, and enabling these traits, it is possible to improve hypertension management and enhance overall community health outcomes.

## Data Availability

The IFLS-5 datasets are available for access after registering on the RAND Corporation's website (https://www.rand.org/well-being/social-and-behavioral-policy/data/FLS/IFLS.html). The geospatial vector data is available from the GADM’s website (https://gadm.org/).

## Abbreviations

AIC: Akaike’s information criterion
CHVs: Community health volunteers
GWR: Geographically weighted regression
IFLS-5: The fifth wave of the Indonesian Family Life Survey
LMICs: Low- and middle-income countries
OLS: Ordinary least squares model
SAR: Spatial autoregressive model
SEM: Spatial error model
SLM: Spatial lag model

## References

[1] NCD Risk Factor Collaboration (NCD-RisC). Worldwide trends in blood pressure from 1975 to 2015: a pooled analysis of 1479 population-based measurement studies with 19.1 million participants. Lancet. 2017. 10.1016/S0140-6736(16)31919-5. 10.1016/S0140-6736(16)31919-5 PMC5220163 27863813

[2] NCD Risk Factor Collaboration (NCD-RisC). Worldwide trends in hypertension prevalence and progress in treatment and control from 1990 to 2019: a pooled analysis of 1201 population-representative studies with 104 million participants. Lancet. 2021. 10.1016/S0140-6736(21)01330-1. 10.1016/S0140-6736(21)01330-1 PMC8446938 34450083

[3] Kementerian Kesehatan Rebuplik Indonesia [Indonesian Ministry of Health]. Riset Kesehatan Dasar (RISKESDAS) 2013 [Basic Health Research 2013]. 2013. https://repository.badankebijakan.kemkes.go.id/id/eprint/4467/1/Laporan_riskesdas_2013_final.pdf. Accessed 3 Mar 2025.

[4] Kementerian Kesehatan Republik Indonesia [Indonesian Ministry of Health]. Laporan tematik Survei Kesehatan Indonesia tahun 2023 [Thematic report on the Indonesian Health Survey 2023]. 2024. https://www.badankebijakan.kemkes.go.id/laporan-tematik-ski/. Accessed 13 Mar 2025.

[5] Cappuccio FP, Kerry SM, Forbes L, Donald A. Blood pressure control by home monitoring: meta-analysis of randomised trials. BMJ. 2004. 10.1136/bmj.38121.684410.AE. 15194600 10.1136/bmj.38121.684410.AE PMC478224

[6] Bray EP, Holder R, Mant J, McManus RJ. Does self-monitoring reduce blood pressure? Meta-analysis with meta-regression of randomized controlled trials. Ann Med. 2010. 10.3109/07853890.2010.489567. 20504241 10.3109/07853890.2010.489567

[7] Glynn LG, Murphy AW, Smith SM, Schroeder K, Fahey T. Interventions used to improve control of blood pressure in patients with hypertension. Cochrane Database Syst Rev. 2010. 10.1002/14651858.CD005182.pub4. 20238338 10.1002/14651858.CD005182.pub4 PMC13248079

[8] Agarwal R, Bills JE, Hecht TJ, Light RP. Role of home blood pressure monitoring in overcoming therapeutic inertia and improving hypertension control: a systematic review and meta-analysis. Hypertension. 2011. 10.1161/HYPERTENSIONAHA.110.160911. 21115879 10.1161/HYPERTENSIONAHA.110.160911

[9] Choi WS, Kim NS, Kim AY, Woo HS. Nurse-coordinated blood pressure telemonitoring for urban hypertensive patients: a systematic review and meta-analysis. Int J Environ Res Public Health. 2021. 10.3390/ijerph18136892. 34199019 10.3390/ijerph18136892 PMC8297065

[10] Geldsetzer P, Manne-Goehler J, Marcus ME, Ebert C, Zhumadilov Z, Wesseh CS, et al. The state of hypertension care in 44 low-income and middle-income countries: a cross-sectional study of nationally representative individual-level data from 1.1 million adults. Lancet. 2019. 10.1016/S0140-6736(19)30955-9. 31327566 10.1016/S0140-6736(19)30955-9

[11] World Health Organization. Hypertension. 2023. https://www.who.int/news-room/fact-sheets/detail/hypertension. Accessed 13 Mar 2025.

[12] Schutte AE, Srinivasapura Venkateshmurthy N, Mohan S, Prabhakaran D. Hypertension in low- and middle-income countries. Circ Res. 2021. 10.1161/CIRCRESAHA.120.318729. 33793340 10.1161/CIRCRESAHA.120.318729 PMC8091106

[13] Mills KT, Stefanescu A, He J. The global epidemiology of hypertension. Nat Rev Nephrol. 2020. 10.1038/s41581-019-0244-2. 32024986 10.1038/s41581-019-0244-2 PMC7998524

[14] World Health Organization Regional Office for Africa. A heavy burden: the productivity cost of illness in Africa. 2019. https://www.afro.who.int/sites/default/files/2019-03/Productivity%20cost%20of%20illness%202019-03-21.pdf. Accessed 13 Mar 2025.

[15] Kementerian Kesehatan Rebuplik Indonesia [Indonesian Ministry of Health] Petunjuk teknis pos pembinaan terpadu penyakit tidak menular (Posbindu PTM) [Technical guidelines for integrated posts for non-communicable diseases]. 2012. http://p2ptm.kemkes.go.id/uploads/2016/10/Petunjuk-Teknis-Pos-Pembinaan-Terpadu-Penyakit-Tidak-Menular-POSBINDU-PTM.pdf. Accessed 3 Mar 2025.

[16] Mizutani M, Tashiro J, Maftuhah. Community health system assessment for noncommunicable disease prevention and health promotion in Indonesia: a nursing perspective. J Shiga Univ Med Sci. 2017;30:17–24.

[17] Mizutani M, Sugiarto H, Bando H, Kondo I, Mock J. Positive deviance: frequent blood pressure monitoring among non-hypertensive middle-aged women in rural Indonesia. Acta Med Indones. 2021;53:397–406. 35027486

[18] Safary E, Mwandeti M, Matanje B, Beiersmann C, Mtaita C, Shiroya V, et al. Role of community health volunteers in identifying people with elevated blood pressure for diagnosis and monitoring of hypertension in Malawi: a qualitative study. BMC Cardiovasc Disord. 2021. 10.1186/s12872-021-02171-7. 34330218 10.1186/s12872-021-02171-7 PMC8325216

[19] Gyawali B, Sharma R, Mishra SR, Neupane D, Vaidya A, Sandbæk A, et al. Effectiveness of a female community health volunteer-delivered intervention in reducing blood glucose among adults with type 2 diabetes: an open-label, cluster randomized clinical trial. JAMA Netw Open. 2021. 10.1001/jamanetworkopen.2020.35799. 33523189 10.1001/jamanetworkopen.2020.35799 PMC7851734

[20] Mbuthia GW, Magutah K, Pellowski J. Approaches and outcomes of community health worker’s interventions for hypertension management and control in low-income and middle-income countries: systematic review. BMJ Open. 2022. 10.1136/bmjopen-2021-053455. 35365519 10.1136/bmjopen-2021-053455 PMC8977767

[21] Kate MP, Samuel C, Singh S, Jain M, Kamra D, Singh GB, et al. Community health volunteer for blood pressure control in rural people with stroke in India: pilot randomised trial. J Stroke Cerebrovasc Dis. 2023. 10.1016/j.jstrokecerebrovasdis.2023.107107. 37003249 10.1016/j.jstrokecerebrovasdis.2023.107107

[22] Tobler WB. A computer movie simulating urban growth in the Detroit region. Econ Geogr. 1970. 10.2307/143141.

[23] Anselin L. Spatial econometrics. In: Baltagi BH, editor. A companion to theoretical econometrics. Oxford: Blackwell; 2003. 10.1002/9780470996249.ch15.

[24] Fotheringham AS, Charlton ME, Brunsdon C. Geographically weighted regression: a natural evolution of the expansion method for spatial data analysis. Environ Plann A Econ Space. 1998. 10.1068/a301905.

[25] Strauss J, Witoelar F, Sikoki B. The fifth wave of the Indonesia Family Life Survey (IFLS5): overview and field report. Volume 1. RAND Labor & Population. 2016. https://www.rand.org/content/dam/rand/pubs/working_papers/WR1100/WR1143z1/RAND_WR1143z1.pdf. Accessed 3 Mar 2025.

[26] GADM. GADM data. Version 4.0. 2022. https://gadm.org/. Accessed 5 May 2022.

[27] Ostchega Y, Berman L, Hughes JP, Chen TC, Chiappa MM. Home blood pressure monitoring and hypertension status among US adults: the National Health and Nutrition Examination Survey (NHANES), 2009–2010. Am J Hypertens. 2013. 10.1093/ajh/hpt054. 23604493 10.1093/ajh/hpt054

[28] Ostchega Y, Zhang G, Kit BK, Nwankwo T. Factors associated with home blood pressure monitoring among US adults: national health and nutrition examination survey, 2011–2014. Am J Hypertens. 2017. 10.1093/ajh/hpx101. 28633432 10.1093/ajh/hpx101 PMC9880871

[29] Wang Q, Xu L, Sun L, Li J, Qin W, Ding G, et al. Rural-urban difference in blood pressure measurement frequency among elderly with hypertension: a cross-sectional study in Shandong, China. J Health Popul Nutr. 2018. 10.1186/s41043-018-0155-z. 30466482 10.1186/s41043-018-0155-z PMC6249846

[30] Kovell LC, Maxner B, Shankara S, Lemon SC, Person SD, Moore Simas TA, et al. Home blood pressure monitoring in women of child-bearing age with hypertension from 2009 to 2014. Am J Hypertens. 2020. 10.1093/ajh/hpac055. 10.1093/ajh/hpac055 PMC9340649 35512277

[31] Tanimura S. Kango kenkyuu ni okeru sample size no kangae kata [Approaches to sample size determination in nursing research]. Mie Nurs J. 2023;25:59–70.

[32] Cohen J. Statistical power analysis for the behavioral sciences. 2nd ed. Hillsdale: Lawrence Erlbaum; 1988.

[33] Bivand RS, Pebesma E, Gómez-Rubio V. Applied spatial data analysis with R. 2nd ed. New York City: Springer; 2013.

[34] R Core Team. R: a language and environment for statistical computing. R Foundation for Statistical Computing. 2024. https://www.R-project.org/. Accessed 21 Sep 2024.

[35] Bivand R, Piras G. spatialreg: Spatial Regression Techniques (Version 1.3–6) [R package]. 2024. https://CRAN.R-project.org/package=spatialreg. Accessed 5 May 2025.

[36] Bivand R, Yu D. spgwr: Geographically Weighted Regression (Version 0.6–37). [R package]. 2024. https://CRAN.R-project.org/package=spgwr. Accessed 5 May 2025.

[37] Direktorat Jenderal Pengendalian Penyakit dan Penyehatan Lingkungan, Kementerian Kesehatan Republik Indonesia [Directorate General of Disease Control and Environmental Health, Indonesian Ministry of Health]. Profil pengendalian penyakit dan penyehatan lingkungan tahun 2011 [Profile of disease control and environmental health 2011]. 2012. https://p2p.kemkes.go.id/wp-content/uploads/2023/12/profilditjenppdanpl2012-130117213544-phpapp01.pdf. Accessed 12 Feb 2025.

[38] World Health Organization. WHO technical specifications for automated non-invasive blood pressure measuring devices with cuff. 2020. https://iris.who.int/bitstream/handle/10665/331749/9789240002654-eng.pdf. Accessed 13 Mar 2025. 10.1161/HYPERTENSIONAHA.120.16625 PMC7884242 33517681

[39] Wang JG, Bunyi ML, Chia YC, Kario K, Ohkubo T, Park S, et al. Insights on home blood pressure monitoring in Asia: expert perspectives from 10 countries/regions. J Clin Hypertens (Greenwich). 2021. 10.1111/jch.14074. 33043574 10.1111/jch.14074 PMC7891443

[40] Elnaem MH, Mosaad M, Abdelaziz DH, Mansour NO, Usman A, Elrggal ME, et al. Disparities in prevalence and barriers to hypertension control: a systematic review. Int J Environ Res Public Health. 2022. 10.3390/ijerph192114571. 36361453 10.3390/ijerph192114571 PMC9655663

[41] Yangsen BR, Lewa I, Badaruddin MS. The shift of character and role of Minangkabau women in novel *Perempuan Batih* by A.R. Rizal. ELS J Interdiscipl Stud Hum. 2021. 10.34050/elsjish.v4i3.17963.

[42] Bhanbhro S, Kamal T, Diyo RW, Lipoeto NI, Soltani H. Factors affecting maternal nutrition and health: a qualitative study in a matrilineal community in Indonesia. PLoS ONE. 2020. 10.1371/journal.pone.0234545. 32544180 10.1371/journal.pone.0234545 PMC7297355

[43] Lipoeto NI, Mmedsci AZ, Oenzil F, Masrul M, Wattanapenpaiboon N. Contemporary Minangkabau food culture in West Sumatra, Indonesia. Asia Pac J Clin Nutr. 2001. 10.1046/j.1440-6047.2001.00201.x. 11708602 10.1046/j.1440-6047.2001.00201.x

[44] Nur SM, Rasminto, Khausar. Pendidikan karakter dalam perspektif kebudayaan (studi pada keluarga suku Batak Toba) [Character education from a cultural perspective (a study on the Batak Toba family)]. Bina Gogik. 2019;6(2):61–74.

[45] Hartley M, Repede E. Nurse practitioner communication and treatment adherence in hypertensive patients. J Nurse Pract. 2011. 10.1016/j.nurpra.2011.04.017.

[46] Badan Pusat Statistik Indonesia [Central Bureau of Statistics Indonesia]. Population density by province (person/sq.km), 2021. 2023. https://www.bps.go.id/en/statistics-table/2/MTQxIzI=/population-density-by-province.html. Accessed 3 Mar 2025.

[47] Badan Pusat Statistik Indonesia [Central Bureau of Statistics Indonesia]. Statistik Indonesia 2025 [Indonesia Statistics 2025]. 2025. https://www.bps.go.id/id/publication/2025/02/28/8cfe1a589ad3693396d3db9f/statistik-indonesia-2025.html. Accessed 27 May 2025.

[48] Kementerian Kesehatan Republik Indonesia [Indonesian Ministry of Health]. Dokumen Target Rasio Tenaga Kesehatan Tahun 2022 [Health Workforce Ratio Target Document 2022]. 2023. https://pusatkrisis.kemkes.go.id/dokumen-target-rasio-tenaga-kesehatan-tahun-2022. Accessed 27 May 2025.

[49] Badan Kependudukan dan Keluarga Berencana Nasional [National Population and Family Planning Board] Pelatihan kader posyandu [Community health volunteer training]. 2024. https://kampungkb.bkkbn.go.id/kampung/38175/intervensi/1114514/pelatihan-kader-posyandu. Accessed 28 Feb 2025.

[50] Kementerian Kesehatan Republik Indonesia [Indonesian Ministry of Health]. Pelatihan keterampilan dasar kader posyandu tahun 2024 angkatan II [Basic skills training for community health volunteers in 2024 Batch II]. 2024. https://lms.kemkes.go.id/courses/29737be1-04af-415d-9717-da5c8d644d24. Accessed 28 Feb 2025.

[51] Wakefield J. Ecologic studies revisited. Annu Rev Public Health. 2008. 10.1146/annurev.publhealth.29.020907.090821. 17914933 10.1146/annurev.publhealth.29.020907.090821

